# Trends in Urinary Sodium-to-Potassium Ratios in Koreans: Analysis of KNHANES 2016–2023 Data

**DOI:** 10.3390/nu17152411

**Published:** 2025-07-24

**Authors:** Rihwa Choi, Gayoung Chun, Sung-Eun Cho, Sang Gon Lee

**Affiliations:** 1 Laboratory Medicine Center, Division of Laboratory Medicine, GC Labs, Yongin-si 16924, Republic of Korea; pirate0720@naver.com; 2 Department of Laboratory Medicine and Genetics, Sungkyunkwan University School of Medicine, Seoul 06351, Republic of Korea; 3 Biostatistics Team, Infectious Disease Research Center, Division of Laboratory Medicine, GC Labs, Yongin-si 16924, Republic of Korea; forjund@gclabs.co.kr; 4 Endocrine Substance Analysis Center (ESAC), Division of Laboratory Medicine, GC Labs, Yongin-si 16924, Republic of Korea; secho1206!!@gclabs.co.kr

**Keywords:** sodium, potassium, urinary electrolytes, KNHANES, nutritional epidemiology

## Abstract

**Background/Objectives**: Recent Japanese guidelines recommend using the average sodium-to-potassium (Na/K) ratio from casual urine samples to assess hypertension and cardiovascular risk, suggesting cutoffs of 2 (optimal) and 4 (feasible). We aimed to evaluate the proportion of Korean individuals who would be classified as having elevated Na/K ratios using these cutoffs, based on random urine Na/K measurements obtained from the nationally representative Korea National Health and Nutrition Examination Survey (KNHANES) dataset. **Methods**: We analyzed 50,440 participants from the KNHANES 2016–2023 with available random urine Na and K results. Annual urinary Na/K ratios were calculated, and the prevalence of ratios ≥2 and ≥4 was assessed by age and sex using sampling weights. **Results**: The weighted median Na/K ratios were consistently lower than the weighted means, indicating skewed distributions. From 2016 to 2023, the weighted median and mean values increased from 2.3 and 2.7 to 2.8 and 3.3, respectively. The prevalence of a Na/K ratio ≥2 increased from 60.5% to 72.0%, and that of a ratio ≥4 increased from 16.9% to 28.3%. A U-shaped trend in Na/K ≥4 prevalence was observed by age, highest among those <20 and ≥70 years. Males had a higher prevalence than females in all age groups except 20–29. **Conclusions**: A growing proportion of Koreans exceeded Na/K cutoffs of 2 and 4 over time. Age- and sex-specific variation suggests tailored interpretation may be necessary when applying these thresholds in population health monitoring.

## 1. Introduction

Electrolytes such as sodium (Na) and potassium (K) play essential roles in maintaining blood pressure homeostasis and fluid balance [1]. Urinary excretion of these electrolytes reflects both dietary intake and renal regulation, and is typically assessed using 24 h urine collection to account for diurnal variation and total daily excretion [2]. However, 24 h urine collection is logistically challenging and susceptible to collection errors, leading to growing interest in the use of random (spot) urine samples as a surrogate [2,3,4].

Several population-based studies worldwide have explored the utility of the urinary Na/K ratio derived from spot urine samples as a biomarker for cardiovascular risk, including hypertension and stroke [2,3,4,5,6,7]. These studies have demonstrated not only the prognostic value of the urinary Na/K ratio, but also revealed substantial interethnic differences in baseline values and risk thresholds [2]. Although a Na/K ratio below 1 has been proposed as an optimal target in global studies, researchers emphasize the importance of population-specific validation due to dietary and genetic variability [2]. In response to this need, the Working Group of the Japanese Society of Hypertension recently issued a consensus statement recommending population-based Na/K ratio thresholds for cardiovascular risk stratification, based on repeated spot urine measurements and validation with 24 h collections [2]. They suggested that a Na/K ratio below 2 be considered optimal, and a value below 4 acceptable (feasible) for assessing hypertension risk in healthy populations [2].

The Korea National Health and Nutrition Examination Survey (KNHANES) is a nationally representative, large-scale, cross-sectional study that provides comprehensive data on the health and nutritional status of the Korean population [8,9]. Conducted by the Korea Disease Control and Prevention Agency (KDCA), the KNHANES has served as a cornerstone resource for epidemiological research and health policy development in Korea [8]. Despite the availability of extensive health and nutrition data from the KNHANES, studies focusing specifically on the urinary Na/K ratio in the Korean population remain limited [10].

Therefore, the aim of this study was to retrospectively analyze random urine sodium and potassium data from the KNHANES to characterize the distribution of urinary Na/K ratios in the Korean population. The findings may enhance the understanding of electrolyte excretion patterns among Koreans, support population-specific cutoff recommendations for cardiovascular risk stratification, and underscore the utility of open-access public health datasets in generating evidence-based clinical insights.

## 2. Materials and Methods

This retrospective cross-sectional study utilized data from the KNHANES conducted between 2016 and 2023. The KNHANES is an annual, nationwide health survey administered by the KDCA, employing a stratified, multistage probability sampling design to ensure representativeness of the Korean population. All datasets are publicly available and de-identified for research purposes. Participants were selected from survey cycles in which random urine Na and K concentrations were measured. Individuals with missing urinary Na or K data were excluded. Spot urine samples were collected as part of the health examination component. Urinary Na and K concentrations (mmol/L) were measured using the ion-selective electrode (ISE) method. The urinary Na/K ratio was calculated for each participant as the ratio of urine Na to K (mmol/L).

Due to differences in analyzers, testing laboratories, and reportable ranges of urinary sodium and potassium across survey phases, only values falling within the overlapping reportable ranges were included in cross-year comparisons. According to the KNHANES data transformation protocol, urinary Na values reported as <20 mmol/L were imputed as 14.1 mmol/L (i.e., 20 divided by √2), and urinary K values reported as <3 mmol/L were imputed as 2.1 mmol/L (i.e., 3 divided by √2) [8,11,12,13,14,15,16]. Similarly, values reported as >250 mmol/L for Na and >100 mmol/L for K were truncated at 251 mmol/L and 101 mmol/L, respectively [8]. Participants were categorized into 10-year age groups (<20 years, 20–29, 30–39, …, ≥70 years), and stratified descriptive statistics were generated for each combination of survey year, age group, and sex.

Descriptive statistics were used to summarize urine sodium, potassium, and the Na/K ratio values by year, sex, and age group. The normality of continuous variables was assessed using the Kolmogorov–Smirnov test, given the large sample size. Distributions of the Na/K ratio across survey years were visualized using kernel density plots. The proportions of individuals with Na/K ratios <1, <2, and <4 were calculated annually to evaluate alignment with internationally proposed cardiovascular risk thresholds [2].

All statistical analyses were conducted using complex sample design methods in accordance with the official statistical analysis guidelines of the KNHANES [8]. Sampling weights, strata, and primary sampling units provided by the survey were incorporated using Taylor series linearization for variance estimation [17]. To evaluate temporal trends in urinary Na, K, and the Na/K ratio, generalized linear models (GLM) accounting for the complex sampling design were fitted. To examine associations between demographic variables and elevated urinary Na/K ratio (defined as ≥4), survey-weighted logistic regression models were used. Because survey-weighted models estimate standard errors based on the sampling design rather than relying on distributional assumptions, assessment of residual normality was not required [17]. Statistical programming and data visualization were supported by generative AI tools (ChatGPT) to enhance reproducibility and efficiency. Statistical analyses were conducted using R version 4.3.2 (R Foundation for Statistical Computing, Vienna, Austria). All final analyses and interpretations were manually validated. Statistical significance was defined as *p* < 0.05.

## 3. Results

We included all available data from the 2016–2023 KNHANES cycles, as only quality-assured specimens are released following internal validation via the KNHANES. Of the 60,022 individuals who participated in the KNHANES from 2016 to 2023, 50,440 participants with available urine sodium and potassium measurements were included in the analysis (weighted mean age, 45.1 years; SD, 18.7; 49.9% female).

Over the 8-year period, the distribution of age, sex, and other basic demographic characteristics remained relatively stable, although a modest increase in mean age was observed—from 2016 to 2023, the average age rose by approximately 3.4 years (Table 1).

**Figure 1:**
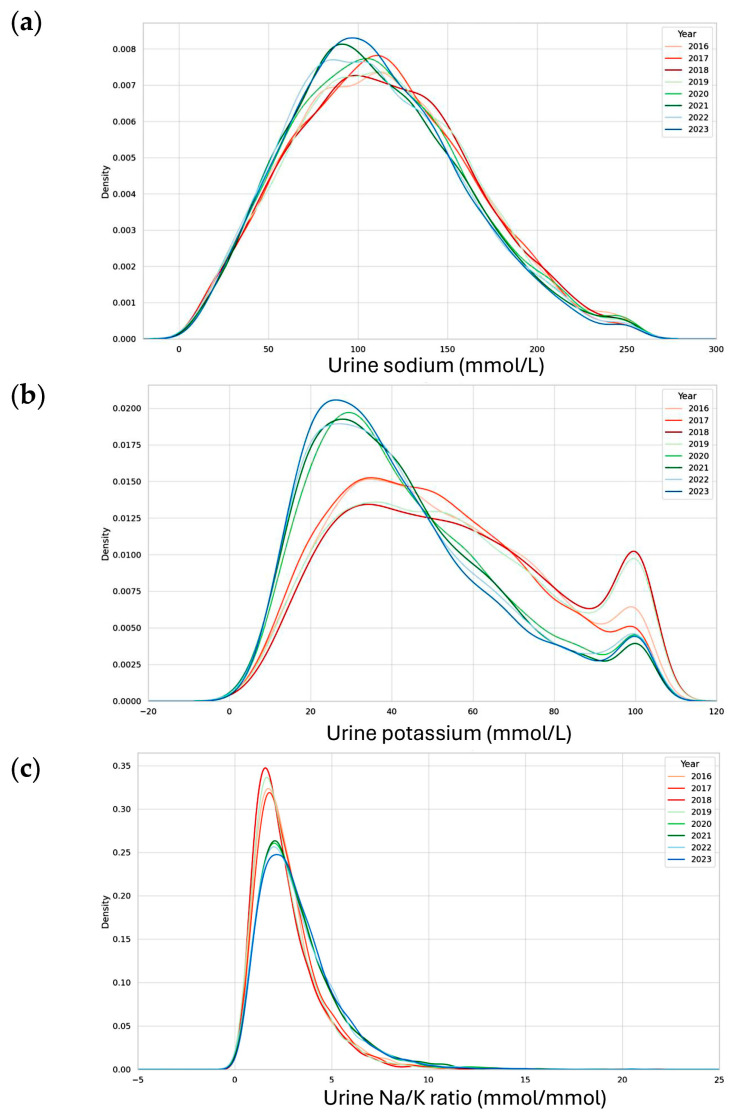
Urinary sodium, potassium, and their ratio (Na/K ratio) in KNHANES 2016–2023. (**a**) Kernel density plots of urinary sodium (**top**), urinary potassium (**middle**) (**b**), and urine Na/K ratio (**bottom**) (**c**). Each KNHANES phase was represented using a consistent color hue, with darker shades indicating more recent survey years within the same phase. KNHANES Phase VII (2016–2018) was depicted in shades of red, Phase VIII (2019–2021) in shades of green, and Phase IX (2022–2023) in shades of blue.

Kernel density plots were generated to visualize the annual distributions of urinary Na, K, and the Na/K ratio (Figure 1). A notable change was observed beginning in KNHANES Phase VIII (2020), when the testing laboratory, Seegene Medical Foundation, implemented a change in the manufacturer of the urinary electrolyte assay in 2018. Although the impact on urinary Na levels was relatively modest, the kernel density plots revealed a visibly distinct shift in the distribution of urinary K starting in 2020. A subsequent change in the testing laboratory occurred in 2022; however, the altered distributional pattern persisted through 2023.

Among the analytes examined, urinary K showed the most prominent change in distribution, which in turn influenced the shape of the Na/K ratio distribution curves during the corresponding years. These alterations in urinary Na and K concentrations were also reflected in the Na/K ratio plots, which exhibited similar inflection patterns beginning in 2020 and continuing through 2023.

**Figure 2:**
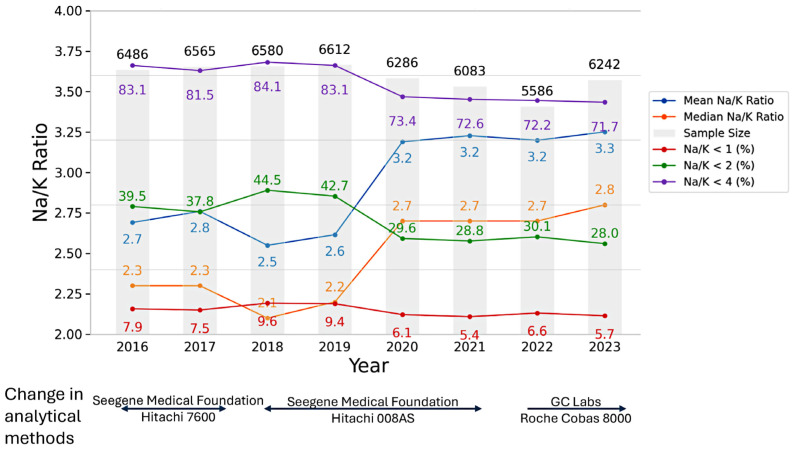
Annual trends in the number and mean age of participants, mean and median urine Na/K ratio, and the prevalence of urine Na/K ratio <1, <2, and <4 (mmol/mmol) from 2016 to 2023.

**Figure 3:**
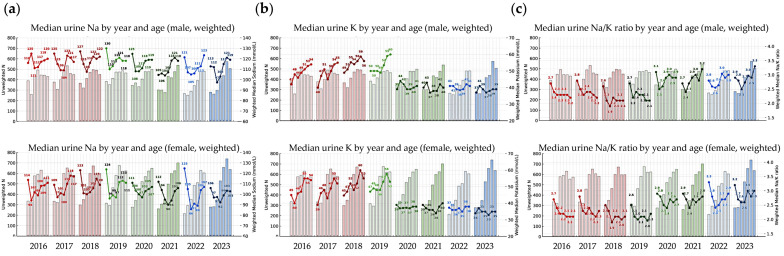
Median urinary sodium (Na), potassium (K), and Na/K ratio, along with the number of participants by sex, age group, and year. (**a**) Median urinary sodium concentration; (**b**) median urinary potassium concentration; and (**c**) median urinary Na/K ratio. Line graphs represent the median urinary Na, K, and Na/K ratio stratified by sex and age group for each year from 2016 to 2023 (males in the **top** panels, females in the **bottom**). Bar graphs in the background indicate the number of participants (n) in each corresponding age group and year.

The annual distributions of test results and participant characteristics—including sex, age, urinary Na, K, and the Na/K ratio—along with information on testing laboratories and measurement instruments from 2016 to 2023, are summarized in Figure 2 and Figure 3. Across all survey years, the median Na/K ratio was consistently lower than the mean, indicating a right-skewed distribution. Except for the years 2018–2019, the median Na/K ratio showed a slight upward trend over time.

Based on the combined dataset from 2016 to 2023, the lowest median Na/K ratio was observed in females (2.1 in 2018), while the highest was observed in males (2.9 in 2023). When stratified by year, the lowest annual median Na/K ratio was observed in 2018 among females and males aged 30–39 years (both 1.9), while the highest was observed in 2022 among females aged <20 years and in 2023 among males aged ≥70 years (both 3.3). From 2016 to 2023, the weighted median and mean Na/K ratios increased from 2.3 and 2.7 to 2.8 and 3.3, respectively.

**Figure 4:**
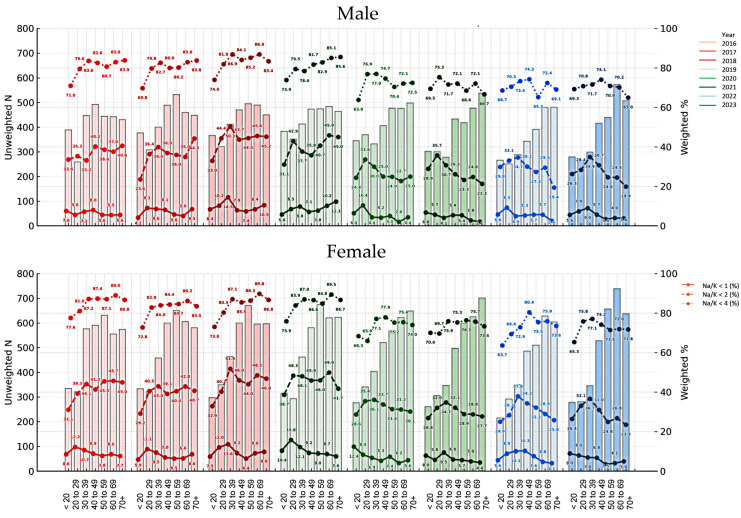
Prevalence of urinary sodium-to-potassium (Na/K) ratios <1, <2, and <4 by age group, sex, and survey year with participant counts. Bar graphs in the background indicate the number of participants (n) in each corresponding age group and year, with darker colors corresponding to more recent survey years within each KNHANES phase. The line graphs are shown as a reference for Na/K thresholds in 2016: dark for <1 (bottom), medium for <2 (middle), and light for <4 (top), illustrating the relative intensity used for all survey years. Bar graphs in the background show the number of participants in each age group and year (left y-axis).

**Figure 5:**
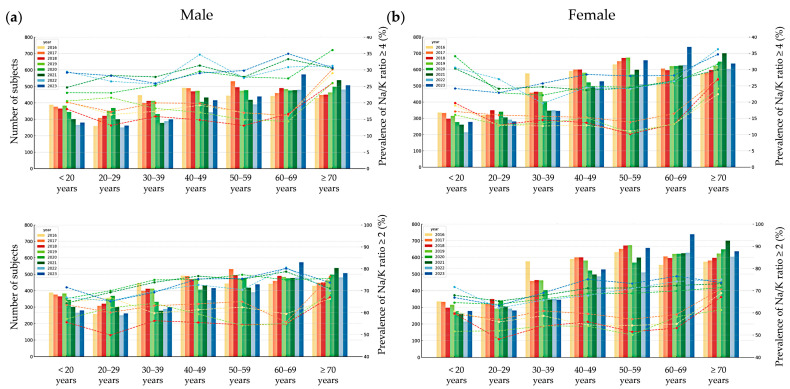
Prevalence of urinary sodium-to-potassium (Na/K) ratios ≥ 4 (top) and ≥ 2 (bottom) by age group and survey year with participant counts in male (**a**) and female (**b**) subjects. Bar graphs in the background indicate the number of participants (n) in each corresponding age group and year (left y-axis).

When applying Na/K ratio thresholds of <1, <2, and <4, a decreasing trend in the prevalence of each category was observed over time, with the exception of 2018 and 2019. From 2016 to 2023, the proportion of participants with Na/K ratios below these thresholds gradually declined, while the proportion with Na/K ratios ≥4 appeared to increase over the same period. The prevalence of Na/K ratio thresholds stratified by sex and age group is presented in Figure 4 and Figure 5. The prevalence of a Na/K ratio ≥2 increased from 60.5% to 72.0%, and that of a ratio ≥4 increased from 16.9% to 28.3%. A U-shaped trend in Na/K ≥4 prevalence was observed by age, highest among those <20 and ≥70 years. Males had a higher prevalence than females in all age groups except 20–29.

After adjusting for age and sex, calendar year was found to be significantly associated with all three urinary electrolyte parameters. The urinary sodium concentration showed a statistically significant decrease over time (β = –0.82, 95% CI: –1.07 to –0.57, *p* < 0.001), and urinary potassium also declined (β = –1.81, 95% CI: –1.96 to –1.67, *p* < 0.001), based on a GLM incorporating the complex sampling design. In contrast, the Na/K ratio increased over the same period (β = +0.11, 95% CI: 0.10 to 0.12, *p* < 0.001). Age was positively associated with both Na and K levels, but showed a slight inverse association with the Na/K ratio (β = –0.004, *p* < 0.001). Male sex was independently associated with higher urinary Na, K, and the Na/K ratio compared to female participants (all *p* < 0.001).

**Figure 6:**
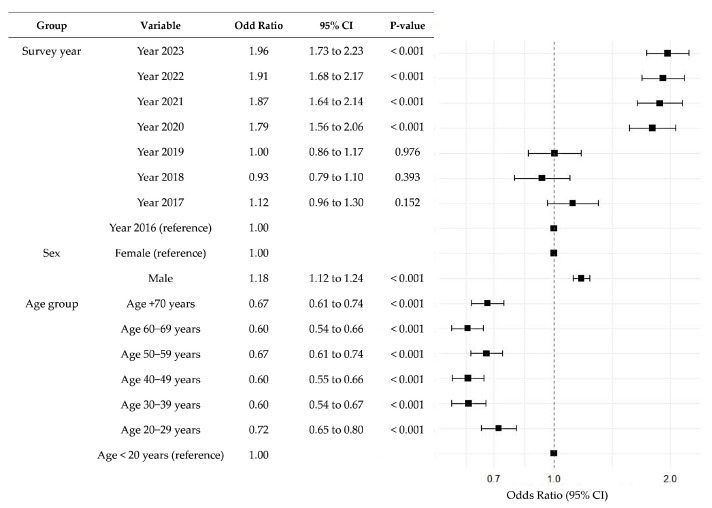
Multiple logistic regression analysis for factors associated with urinary sodium-to-potassium (Na/K) ratios ≥ 4. This forest plot illustrates the adjusted odds ratios and 95% confidence intervals (CIs) for predictors of having sodium-to-potassium (Na/K) ratios ≥ 4, based on a generalized linear model for complex surveys. The size of each square corresponds to the absolute value of the logarithmic odds ratio, indicating the relative weight of each variable in the model.

Since the prevalence of a Na/K ratio ≥2 was observed in more than half of the participants, a logistic regression analysis using a GLM with a complex sampling design was conducted to investigate factors associated with a high Na/K ratio (≥4), which is considered a feasible target according to the Japanese guidelines (Figure 6). Age group, survey year, and sex were found to be significantly associated with having a Na/K ratio ≥4.

## 4. Discussion

In this study, we analyzed the temporal trends and demographic patterns of urinary Na, K, and the Na/K ratio using nationally representative data from the 2016–2023 KNHANES.

As emphasized by the World Health Organization (WHO) in its recommendations on sodium intake and the reduction in hypertension and cardiovascular disease risk, there is a global effort—including in Korea—to mitigate the burden of cardiovascular diseases [1,18]. The urinary Na/K ratio reflects not only dietary intake, but also various physiological and lifestyle factors, and its distribution varies substantially across populations [2]. For example, an analysis using data from the Multi-Ethnic Study of Atherosclerosis (MESA) demonstrated that a urinary Na/K ratio of ≤1, as measured by spot urine samples, was associated with a 40–50% lower risk of stroke [6]. However, due to population-specific differences in diet, genetics, and environmental exposures, the applicability of universal cutoffs is limited [2]. In recognition of this, the Japanese Society of Hypertension proposed revised Na/K ratio thresholds in its 2024 guidelines, suggesting a value <2 as optimal and <4 as feasible for cardiovascular risk assessment in Japanese populations [2].

Although the WHO recommends limiting daily salt intake to less than 5 g (equivalent to 2000 mg of sodium), most Koreans are reported to consume significantly more than this amount [8,9,10]. The mean and median urinary Na/K ratios observed in the present study far exceeded the ideal cutoff of 1, potentially reflecting the high Na and relatively low K intake patterns prevalent in the Korean population.

Previous studies based on multi-ethnic general populations aged 20–59 years from 32 countries (52 population groups), including 10,079 individuals—of whom 1578 were East Asians and 198 were Koreans from Busan—reported casual urine Na/K ratios as part of the International Study on Salt and Blood Pressure (INTERSALT) [5,6]. In the INTERSALT study, the global prevalence of a high Na/K ratio (≥2 and ≥4) was 60.0% and 21.4%, respectively, which is comparable to the findings of the present study [5,6]. The mean Na/K ratios reported across the 52 population groups were derived from relatively small sample sizes (ranging from 157 to 200 participants per group). Specifically, the 198 Korean participants from Busan had a mean (SD) Na/K ratio of 3.81 (1.87), which was slightly higher than that observed in our study population, but still consistent with the high prevalence of elevated Na/K ratios reported among Asian populations [6].

The urinary Na/K ratio is known to exhibit high intra-individual variability and is influenced by several factors, including sex, marital status (particularly men living with a spouse), body mass index, renal function, use of antihypertensive medications, fasting duration, season, educational attainment, and nocturnal urination patterns [2]. In the present study, men showed a higher prevalence of elevated Na/K ratios (≥4) compared to women, consistent with findings from previous research. However, other potential influencing factors were not assessed in this study. Future research is needed to clarify the impact of these multiple determinants on the urinary Na/K ratio and to better understand the mechanisms underlying interindividual variability.

It is noteworthy that a high prevalence of a Na/K ratio ≥4 was observed in both younger age groups (<20 years) and older age groups in the present study population. Most previous studies on hypertension and/or cardiovascular disease risk have focused on adults aged over 20 years [4,5,6]. A recent study conducted in 457 healthy Japanese adolescents (aged 12–15 years) reported a mean urinary Na/K ratio of 4.99 ± 2.76, with no significant difference between boys and girls [19]. These values were higher than those observed in Japanese adults included in the INTERSALT study, and this finding is consistent with the results of the present study [4,5,18].

Interestingly, the distribution of dietary sodium intake by age in the Korean population, as reported in the KNHANES, showed the highest intake in individuals aged 40–49 years, with lower intakes in both younger and older age groups, forming a reverse U-shaped pattern [7]. This contrasts with our finding that the prevalence of a high urinary Na/K ratio (≥4) was most prominent among the youngest and oldest age groups. According to the Korea Hypertension Fact Sheet 2024, the crude prevalence of hypertension among adults aged 20 years and older increased from 25.1% (men 28.5%, women 22.1%) in 1998 to 30.1% (men 33.4%, women 26.8%) in 2022 [20]. These trends are similar to the increasing prevalence of a high Na/K ratio (≥4) observed in our study [8,18,20]. Meanwhile, data from the National Health Statistics Plus (KNHANES) indicated that dietary sodium intake in the Korean population has gradually decreased, with individuals aged 30–64 years consuming more sodium than other age groups [21]. This suggests that factors beyond dietary intake may influence urinary Na/K ratios across different age groups [2]. Variations in hydration status, physical activity patterns, hormonal and renal function, and the use of antihypertensive medications may contribute to altered sodium and potassium excretion in individuals under 20 and over 70 years of age, potentially leading to transient increases in urinary Na/K ratios [2,22].

In the present study, the measurement device for urinary electrolytes was changed in 2018, while remaining within the same testing institution, and both the testing laboratory and measurement equipment were changed again in 2022 [8]. There was no substantial difference in urinary electrolyte results between the periods 2016–2019 and 2020–2023 in relation to the assay change. Rather, noticeable changes began to emerge, starting in 2020. Following the outbreak of COVID-19 in late 2019, public health measures in Korea, including self-quarantine and social distancing, led to substantial lifestyle changes. These shifts may have influenced dietary habits and fluid intake, potentially contributing to the observed urinary electrolyte profiles and the prevalence of high Na/K ratios before and after the pandemic [23]. Further longitudinal studies are warranted to determine whether these patterns persist and to assess their long-term implications for public health.

This study has several limitations. First, although spot urine samples are practical for large-scale epidemiologic studies, they are subject to substantial intra-individual variability and may not accurately reflect daily Na or K intake [2]. Our study is limited in that we analyzed cross-sectional spot urine data from different individuals each year, rather than repeated measurements from the same individuals. Therefore, while our findings provide insight into population-level trends, they cannot account for intra-individual temporal variation [2,4]. The urinary Na/K ratio, in particular, is influenced by the time of collection, recent dietary intake, and various physiological factors [2,4]. The Japanese Society of Hypertension recommends using the average of multiple random urine samples collected on at least four separate days to improve accuracy [2,4]. In addition, interpretation of the Na/K ratio should consider factors such as age, renal function, medication use, seasonal variation, socioeconomic status, and lifestyle behaviors [2]. This study did not account for several of these key variables, including renal function, medication use, and intra-individual lifestyle differences, highlighting the need for future studies with repeated measurements and more comprehensive participant data. Although our analysis focused primarily on age, sex, and calendar year, it is important to acknowledge that secular changes in other population characteristics—such as increasing rates of obesity, urbanization, and socioeconomic shifts—may also contribute to changes in urinary Na/K ratios. For example, rising obesity prevalence and dietary westernization in Korea may be associated with increased Na intake and reduced K consumption [2,4,6,21]. Similarly, regional differences in dietary patterns and access to fresh produce may affect Na and K excretion [2,4,6]. Future studies should explore these associations by incorporating BMI, dietary data, and socioeconomic indicators to provide a more comprehensive understanding of Na/K ratio dynamics over time. Second, changes in assay manufacturers and testing laboratories over time may have introduced measurement variability—particularly in urinary K—as reflected in the kernel density plots [24,25]. In the present study, imputation for values below the lower limit of quantification and above the upper limit of quantification was applied based on differences in analytical methods to calculate the urinary Na/K ratio for annual trend analysis and comparison. As such imputations can introduce bias, caution is warranted. When an assay method is changed, the KNHANES typically provides statistical analysis guidelines for researchers, and, when necessary, publishes conversion equations [8,11,12,13,14,15,16,24]. For urinary Na and K, method comparison was conducted in accordance with CLSI guidelines before and after the assay method change by the separate Quality Control Committee of the Clinical Laboratory for the Korea National Health and Nutrition Examination Survey [8,11,12,13]. According to the official report, the results were deemed acceptable, and no conversion equation was provided [8,11,12,13]. Since there is no universally mandated guideline for handling such data in statistical analyses, researchers should be aware that the choice of statistical approach may influence the results. Despite these limitations, the large sample size and standardized survey methodology of the KNHANES provide robust population-level insights into electrolyte trends and laboratory-based variability.

## 5. Conclusions

This study provides the first comprehensive temporal analysis of urinary Na, K, and the Na/K ratio using 8 years of KNHANES data. A substantial proportion of the Korean population exceeded the proposed urinary Na/K ratio thresholds of 2 and 4, with an increasing trend over time. The observed distribution patterns varied by age and sex, underscoring the need for demographic-specific interpretation when applying these cutoffs for hypertension and cardiovascular risk assessment in public health surveillance.

## Figures and Tables

**Table 1:** Characteristics of KNHANES 2016–2023 data for this study.

Survey Characteristics						Participants			Urine Analyte		
Survey Phase	Laboratory	Methods	Instrument	Reportable Range of Urine Electrolytes	Year	*n*	% of Female	Age (Mean ± SD)	Na (mmol/L)	K (mmol/L)	Na/K Ratio
VII (2016~2018) ^a^	Seegene Medical Foundation	Indirect (diluted) ISE	Hitachi Automatic Analyzer 7600 (Hitachi, Japan)	Na: 10–250 mmol/L K: 1–100 mmol/L	2016	6486	49.9	40.4 ± 20.6	112 (76 to 148)	49 (32 to 70)	2.3 (1.5 to 3.3)
					2017	6565	49.9	40.9 ± 20.7	111 (77 to 147)	48 (31 to 67)	2.3 (1.6 to 3.5)
			Labospect 008AS (Hitachi, Japan) ^a^		2018	6580	49.9	41.3 ± 20.7	112 (77 to 149)	53 (34 to 76)	2.1 (1.4 to 3.2)
VIII (2019~2021)	Seegene Medical Foundation	Indirect (diluted) ISE	Labospect 008AS (Hitachi, Japan)	Na: 10–250 mmol/L K: 1–100 mmol/L	2019	6612	49.9	41.8 ± 20.8	111 (77 to 149)	52 (33 to 74)	2.2 (1.5 to 3.3)
					2020	6286	49.9	42.3 ± 20.9	109 (75 to 145)	39 (26 to 58)	2.7 (1.8 to 4.1)
					2021	6083	49.9	42.9 ± 20.9	105 (75 to 143)	38 (25 to 57)	2.7 (1.8 to 4.1)
XI (2022~2023) ^a^	GC Labs	Indirect (diluted) ISE	Cobas 8000 (Roche, Germany)	Na: 20–350 mmol/L K: 3–100 mmol/L	2022	5586	50.0	43.4 ± 20.9	105 (73 to 142)	38 (25 to 57)	2.8 (1.8 to 4.2)
					2023	6242	50.0	43.8 ± 20.9	105 (74 to 140)	37 (24 to 55)	2.8 (1.8 to 4.2)

Abbreviations: ISE, ion-selective electrode method. Data for urine electrolytes and their ratios presented as median and interquartile range since they were non-parametric. All estimates were calculated using sampling weights to account for the complex survey design of KNHANES. ^a^ The analytical method was changed in 2018 and again in 2022.

## Data Availability

This study utilized the KNHANES public database, which is openly available to the public (https://knhanes.kdca.go.kr/knhanes/eng/main.do (accessed on 24 June 2025)).

## Abbreviations

The following abbreviations are used in this manuscript:

K	Potassium
KDCA	Korea Disease Control and Prevention Agency
KNHANES	Korea National Health and Nutrition Examination Survey
Na	Sodium
NHANES	National Health and Nutrition Examination Survey
WHO	World Health Organization

## References

[1] World Health Organization (2023). WHO Global Report on Sodium Intake Reduction. https://iris.who.int/bitstream/handle/10665/366393/9789240069985-eng.pdf?sequence=1.

[2] Hisamatsu T., Kogure M., Tabara Y., Hozawa A., Sakima A., Tsuchihashi T., Yoshita K., Hayabuchi H., Node K., Takemi Y. (2024). Practical use and target value of urine sodium-to-potassium ratio in assessment of hypertension risk for Japanese: Consensus Statement by the Japanese Society of Hypertension Working Group on Urine Sodium-to-Potassium Ratio. Hypertens. Res..

[3] Mirmiran P., Gaeini Z., Bahadoran Z., Ghasemi A., Norouzirad R., Tohidi M., Azizi F. (2021). Urinary sodium-to-potassium ratio: A simple and useful indicator of diet quality in population-based studies. Eur. J. Med. Res..

[4] Koo H., Lee S.-G., Kim J. (2015). Evaluation of random urine sodium and potassium compensated by creatinine as possible alternative markers for 24 hours urinary sodium and potassium excretion. Ann. Lab. Med..

[5] Intersalt Cooperative Research Group (1988). Intersalt: An international study of electrolyte excretion and blood pressure. Results for 24 hour urinary sodium and potassium excretion. BMJ.

[6] Iwahorim T., Miura K., Ueshima H., Tanaka-Mizuno S., Chan Q., Arima H., Dyer A.R., Elliott P., Stamler J., for the INTERSALT Research Group (2019). Urinary sodium-to-potassium ratio and intake of sodium and potassium among men and women from multiethnic general populations: The INTERSALT Study. Hypertens. Res..

[7] Averill M.M., Young R.L., Wood A.C., Kurlak E.O., Kramer H., Steffen L., McClelland R.L., Delaney J.A., Drewnowski A. (2019). Spot Urine Sodium-to-Potassium Ratio Is a Predictor of Stroke. Stroke.

[8] Korea Disease Control and Prevention Agency Korea National Health and Nutrition Examination Survey (KNHANES). https://knhanes.kdca.go.kr/knhanes/eng/main.do.

[9] Oh K., Kim Y., Kweon S., Kim S., Yun S., Park S., Lee Y.-K., Kim Y., Park O., Jeong E.K. (2021). Korea National Health and Nutrition Examination Survey, 20th anniversary: Accomplishments and future directions. Epidemiol. Health.

[10] Yoon Y., Son M. (2024). Association between blood pressure control in hypertension and urine sodium to potassium ratio: From the Korea National Health and Nutrition Examination Survey (2016–2021). PLoS ONE.

[11] Korea Disease Control and Prevention Agency Quality Control of the Clinical Laboratory for the Korea National Health and Nutrition Examination Survey (KNHANES) (2018, 7th Third Year). https://www.prism.go.kr/homepage/asmt/popup/1351000-201900009.

[12] Korea Disease Control and Prevention Agency Quality Control of the Clinical Laboratory for the Korea National Health and Nutrition Examination Survey (KNHANES) (2019–2021, 8th). https://www.prism.go.kr/homepage/asmt/popup/1351000-201900092.

[13] Korea Disease Control and Prevention Agency Quality Control of the Clinical Laboratory for the Korea National Health and Nutrition Examination Survey (2022–2024). https://www.prism.go.kr/homepage/asmt/popup/1790387-202200029.

[14] National Health and Nutrition Examination Survey 2013–2014 Data Documentation, Codebook, and Frequencies. https://wwwn.cdc.gov/Nchs/Data/Nhanes/Public/2013/DataFiles/U1LT_H_R.htm.

[15] (2023). Diabetes mellitus is associated with higher serum neurofilament light chain levels in the general US population. J. Clin. Endocrinol. Metab..

[16] CLSI (2018). Measurement Procedure Comparison and Bias Estimation Using Patient Samples.

[17] Lumley T. (2010). Complex Surveys: A Guide to Analysis Using R.

[18] Park H.K., Lee Y., Kang B.W., Kwon K.I., Kim J.W., Kwon O.S., Cobb L.K., Campbell N.R.C., Blakeman D.E., Kim C.I. (2020). Progress on sodium reduction in South Korea. BMJ Glob. Health.

[19] Zhang Y., Miyai N., Utsumi M., Miyashita K., Arita M. (2024). Spot urinary sodium-to-potassium ratio is associated with blood pressure levels in healthy adolescents: The Wakayama Study. J. Hum. Hypertens..

[20] Kim H.C., Lee H., Lee H.-H., Ahn S.V., Lee J.-M., Cheon D.Y., Jhee J.H., Yoon M., Shin M.-H., Heo J.N. (2025). Korea Hypertension Fact Sheet 2024: Nationwide population-based analysis with a focus on young adults. Clin. Hypertens..

[21] KNHANES National Health Statistics Plus. https://knhanes.kdca.go.kr/knhanes/archive/wsiNationHelthStatsPlus.do.

[22] Campanozzi A., Avallone S., Barbato A., Iacone R., Russo O., De Filippo G., D’Angelo G., Pensabene L., Malamisura B., Cecere G. (2015). High Sodium and Low Potassium Intake among Italian Children: Relationship with Age, Body Mass and Blood Pressure. PLoS ONE.

[23] Choi R., Park W., Chun G., Lee S.G., Lee E.H. (2023). The Utilization of Serum Folate and Homocysteine Tests and the Prevalence of Folate Deficiency in Reproductive-Age Korean Women during the COVID-19 Pandemic. Nutrients.

[24] Choi R., Seo J.D., Cho E.-J., Lee W., Yun Y.-M. (2025). Adjustment Formula for Harmonizing Triglyceride Values in the Korea National Health and Nutrition Examination Survey, 2005–2022. Ann. Lab. Med..

[25] Kim S., Min W.-K. (2025). Toward High-Quality Real-World Laboratory Data in the Era of Healthcare Big Data. Ann. Lab. Med..

